# Regional differences in frailty among older adults with type 2 diabetes: a multicenter cross-sectional study in Japan

**DOI:** 10.1186/s12877-024-05223-7

**Published:** 2024-08-17

**Authors:** Akiko Nishimura, Chie Masuda, Chiyo Murauchi, Miho Ishii, Yuko Murata, Terumi Kawasaki, Mayumi Azuma, Hidenori Arai, Shin-ichi Harashima

**Affiliations:** 1 Department of Chronic Care Nursing, Faculty of Medicine, Kagawa University, 1750-1 Ikenobe, Miki-cho, Kita-gun, Kagawa 761-0793 Japan; 2 Department of Human Health Sciences, Graduate School of Medicine, Kyoto University, 53 Shogoin Kawahara-cho, Sakyo-ku, Kyoto City, Kyoto 606-8507 Japan; 3 Department of Internal Medicine and Diabetes, Goshominami Harashima Clinic, 630 Heinouchi-cho, Nakagyo-ku, Kyoto City, Kyoto 604-0884 Japan; 4 Department of Nursing, Asahikawa City Hospital, 1-1-65 Kinsei-cho, Asahikawa City, Hokkaido 070-8610 Japan; 5 Faculty of Nursing and Graduate School of Nursing, Kansai Medical University, 2-3-1 Shinmachi, Hirakata City, Osaka 573-1010 Japan; 6 Jonan branch, Town Home-visit Medical Care Clinic, Gardenia Kamiikedai 101, 1-40-6 Kamiikedai, Ota-ku, Tokyo, 145-0064 Japan; 7 Department of Nursing, Takashima Municipal Hospital, 1667 Katsuno, Takashima City, Shiga 520-1121 Japan; 8 Department of Nursing, Sapporo City General Hospital, 1-1 Kita, 11-jo Nishi, 13-chome, Chuo- ku, Sapporo City, Hokkaido 064-0064 Japan; 9 Department of Internal Medicine, Miyazaki Prefectural Miyazaki Hospital, 5-30 Kitatakamatsu- cho, Miyazaki City, Miyazaki 880-8510 Japan; 10 National Center for Geriatrics and Gerontology, 7-430 Morioka-cho, Obu City, Aichi 474-8511 Japan; 11 Clinical Research Planning and Administration Division, National Hospital Organization, Kyoto Medical Center, 1-1 Fukakusa Mukabatake-cho, Fushimi-ku, Kyoto City, Kyoto 611-8555 Japan; 12 Research Center for Healthcare, Nagahama City Hospital, 313 Ohinui-cho, Nagahama City, Shiga 526-8580 Japan

**Keywords:** Frailty, Geriatric health care, Type 2 diabetes, Aging issues, Regional differences

## Abstract

**Background:**

Social environment may broadly impact multifaceted frailty; however, how environmental differences influence frailty in older adults with diabetes remains unclear. This study aimed to investigate regional differences in frailty in urban and rural areas among older adults with diabetes.

**Methods:**

This cross-sectional study was conducted as part of the frailty prevention program for older adults with diabetes study. Older adults aged 60–80 years who could independently perform basic activities of daily living (ADLs) were enrolled sequentially. Trained nurses obtained patient background, complications, body weight, body composition, blood tests, grip strength, frailty assessment, and self-care score results. Regional differences in frailty were evaluated using logistic and multiple linear regression analyses.

**Results:**

This study included 417 participants (269 urban and 148 rural). The prevalence of robustness was significantly lower in rural areas than in urban areas (29.7% vs. 43.9%, *p* = 0.018). Living in rural areas was associated with frailty (odds ratio [OR] 2.55, 95% confidence interval [CI] 1.38–4.71) and pre-frailty (OR 2.10, 95%CI 1.30–3.41). Lower instrumental ADL (B 0.28, standard error [SE] 0.073) and social ADL (B 0.265, SE 0.097) were characteristics of rural residents.

**Conclusions:**

Regional differences in frailty were observed. Older adults with diabetes living in rural areas have a higher risk of frailty owing to a decline in instrumental and social ADLs. Social environment assessment and intervention programs that include communication strategies to enable care and social participation across environments are crucial to the effective and early prevention of frailty.

## Background

Frailty is “a clinical state in which there is an increase in an individual’s vulnerability to dependency and mortality when exposed to a stressor” [1, 2]. Frailty has been associated with mortality, reduced activities of daily living (ADLs), hospitalization, physical limitations, falls, and fractures [3]. It is an intermediate stage between being healthy and needing care [4, 5] and could be treated using appropriate interventions, especially in the early stages [6, 7]. Therefore, early assessments to detect frailty and interventions based on these assessments are required to ensure healthy aging [5, 7, 8].

Diabetes mellitus is one of the most common risk factors for frailty [9–11]. In addition, the combination of diabetes and frailty further worsens prognoses, leading to the development of physical dysfunction and mortality [12–14]. Therefore, identifying diabetes-specific factors that influence the development of frailty in older adults with diabetes is crucial to enabling more effective assessments and interventions based on this unique condition of older adults with diabetes.

The risk factors for frailty include a wide range of demographic, social, clinical, lifestyle, and biological factors [7, 15]. However, most previous studies on frailty have focused on the physical aspects using previously reported assessment methods [16–18]. Few studies have examined the risk of frailty in detail, particularly among older adults with diabetes. Therefore, we previously reported multifaceted risk factors associated with diabetes-specific frailty in older adults who could independently perform ADL [19]. The study showed gender differences in these factors, indicating the need to consider gender in preventive interventions. In addition, diabetes medications also influence frailty, particularly in older adults receiving sodium-glucose co-transporter-2 inhibitors (SGLT2i), in whom frailty has already developed, even though they appear to be healthy. Consequently, it is necessary to pay attention to frailty at the time of drug administration [20, 21].

These studies have evaluated various factors; however, regional differences have not been adequately explored owing to the limitations of the study design. Previous studies have reported the regional incidence of frailty in older adults [22, 23]; nevertheless, no study has examined the regional differences in the risk of frailty and its characteristics among older adults with diabetes. If regional differences in frailty are clarified, more effective diabetes care can be proposed for older adults to prevent frailty and manage diabetes. Therefore, this study aimed to investigate the regional differences in frailty between urban and rural areas among older adults with diabetes using a national multicenter survey.

## Methods

### Study design and participants

This cross-sectional study was conducted as part of the frailty prevention program for older adults with diabetes (The f-PPOD) study. This protocol has been previously reported [21]. The inclusion criteria were the presence of type 2 diabetes, age 60–80 years, and unimpaired basic ADL (Barthel index ≥ 85). The exclusion criteria were certification for long-term care/support needs and presence of cerebrovascular and peripheral artery diseases, paralysis in any part of the body, severe diabetic complications, including macrovascular diseases, comorbidities (heart failure, liver and renal disorders, anemia, malignancy, and dementia), and depression or other psychiatric problems. Information on diabetic complications was collected from medical records for peripheral neuropathy, retinopathy, nephropathy, autonomic neuropathy, peripheral arterial disease, and cardiovascular disease, based on annual tests according to guidelines. The physician in charge diagnosed the severity of diabetic complications according to the severity criteria for each condition.

The study participants were recruited between March 21, 2017, and February 7, 2020, from eight outpatient diabetes clinics in Japan. Since this study was a multi-purpose exploratory study on frailty in older adults with diabetes, the sample size was calculated to include 10% (approximately 400 people) of outpatients who met the eligibility criteria at each hospital and clinic. After trained assessors (certified diabetes educators, certified nurses in diabetes nursing, and a diabetologist) screened medical records for eligibility, all participants were sequentially recruited upon arrival at the outpatient clinics.

### Instrument

#### Participants’ demographic characteristics

Demographic characteristics, including age, gender, academic background, family structure, work status, irregular lifestyle (irregular bedtimes and eating habits), drinking habits, smoking habits, and diabetes medications, were obtained from participants’ medical records and through a self-reported general questionnaire. Body composition was measured using the bioelectrical impedance method (HBF-375; OMRON Healthcare Co., Ltd., Kyoto, Japan).

#### Diabetes-related factors

The duration, treatment, and complications of diabetes and blood test results were obtained from participants’ medical records. Hypoglycemia was confirmed by reviewing self-monitored blood glucose records or hypoglycemic episodes reported via a self-reported questionnaire during the last 3 months. Hypoglycemia was defined as a blood glucose level < 70 mg/dL [24] or the presence of hypoglycemic symptoms that improved with carbohydrate intake. Diabetes self-management performance was measured using the Summary of Diabetes Self-Care Activities Measure (SDSCA) [25]. The higher the subscale mean score, the higher the level of self-care practice.

#### Frailty

Frailty was evaluated using the Kihon Checklist (KCL), which was developed and validated by the Japanese Ministry of Health, Labor, and Welfare and is widely used in Japan [26]. The KCL has been translated into other languages and used in various countries [27]. This comprehensive questionnaire assesses multiple domains, including the physical, psychological, functional, and social statuses of older adults without disabilities. A higher score in each KCL domain indicates a higher risk of requiring support or care. KCL scores of ≥ 8 and ≥ 4 points were defined as frailty and pre-frailty, respectively [26].

#### Physical functions

Grip strength was measured using a digital hand dynamometer (T.K.K.5401; Takei Scientific Instruments Co., Ltd., Niigata Prefecture, Japan). The participants were assessed upright, holding the grip dynamometer such that the second joint of the index finger was at 90°.

### Statistical analysis

The participants were categorized into urban and rural areas based on the location of their hospitals to examine regional differences in frailty. Based on the criteria of the Ministry of Internal Affairs and Communications in Japan, urban areas were defined as regions included in the 23 special wards of Tokyo, the government ordinance-designated cities, the municipalities that are contiguous to these areas, and where at least 1.5% of residents aged ≥ 15 years commute to work or school in these areas [28]. Therefore, an urban area was defined as a central city with a population of > 1 million and surrounding municipalities that commute to the central city for work or school.

Data were expressed as mean ± standard deviation for continuous variables or number (percentage) for categorical variables unless otherwise noted. Student’s t-test was used to compare continuous variables, whereas the chi-square and Fisher’s exact tests were used to compare categorical variables according to region.

Logistic regression analyses were performed to evaluate the relationship between frailty and region, adjusting for demographic factors (age and gender) and the basic background of people with diabetes (Hemoglobin A1c [HbA1c] level and diabetes duration).

Statistical significance was set at *p* < 0.05. Data analyses were performed using SPSS statistics (version 28.0; IBM, Japan).

## Results

### Participants’ characteristics

Among 421 eligible participants, four were excluded because of certification for long-term care needs (*n* = 2), withdrawal of consent (*n* = 1), or incomplete data (*n* = 1). Finally, 417 participants (269 urban [64.5%] and 148 rural [35.5%]) were enrolled and analyzed (Table 1).

**Table 1:** Participants’ demographics

Variables	Urban areas (*n* = 269)	Rural areas (*n* = 148)	*p*
Age (years)	70.6 ± 5.5	69.0 ± 5.2	0.003**
Women	129 (48.0)	64 (43.2)	0.356
Body weight (kg)	62.4 ± 11.9	63.9 ± 11.0	0.204
BMI (kg/m^2^)	24.1 ± 3.6	25.0 ± 4.0	0.020*
Diabetes duration (years)	16.6 ± 10.9	12.0 ± 10.3	< 0.001***
HbA1c (%)	7.33 ± 1.00	7.04 ± 0.91	0.003**
Alb (g/ml)	4.16 ± 0.31	4.30 ± 0.38	< 0.001***
LDL-C (mg/dl)	103.7 ± 28.7	106.2 ± 26.6	0.409
CRE (mg/dl)	0.88 ± 0.33	0.80 ± 0.24	0.008**
eGFR (ml/min./1.73m^2^)	63.1 ± 16.9	70.1 ± 18.9	< 0.001***
Total number of diabetic medicines	2.0 ± 1.2	2.1 ± 1.1	0.733
Insulin treatment	93 (34.6)	28 (18.9)	0.001**
SDSCA (self-management score)			
Diet score	5.0 ± 1.3	4.5 ± 1.7	0.003**
Exercise score	3.7 ± 2.4	3.2 ± 2.4	0.036*
Medication score	6.6 ± 1.2	6.9 ± 0.4	0.001**
Hypoglycemia	77 (28.6)	22 (14.9)	0.002**
Serious Hypoglycemia	12 (4.5)	0 (0.0)	0.005**
Nephropathy	58 (21.6)	7 (4.7)	< 0.001***
Retinopathy	79 (29.4)	17 (11.5)	< 0.001***
Peripheral neuropathy	64 (23.8)	6 (4.1)	< 0.001***
Coronary artery disease	46 (17.1)	17 (11.5)	0.126
Peripheral artery disease	13 (4.8)	0 (0)	0.003**
Drinking habit	86 (32.0)	56 (37.8)	0.226
Smoking habit	29 (10.8)	31 (20.9)	0.005**
Inoccupation	164 (61.0)	85 (57.4)	0.481
Occupation	105 (39.0)	63 (42.6)	
Agriculture	7 (6.7)	6 (9.5)	
Public servant	3 (2.9)	2 (3.2)	
Profession	11 (10.5)	1 (1.6)	
Company Executive / Manager	14 (13.3)	9 (14.3)	
Company employee	8 (7.6)	9 (14.3)	
Part-time job	24 (22.9)	21 (33.3)	
Self-employed	28 (26.7)	15 (23.8)	
Unclear	10 (9.5)	0 (0.0)	
Academic background			0.114
Postgraduate school	14 (5.2)	2 (1.4)	
College	62 (23.0)	26 (17.6)	
Junior college / Technical college	33 (12.2)	18 (12.2)	
High school	113 (42.0)	72 (48.6)	
Junior high school and below	47 (17.5)	30 (20.3)	
Living alone	46 (17.1)	31 (20.9)	0.333
Irregular lifestyle	46 (17.1)	22 (14.9)	0.554

Values in the table are presented as the mean ± standard deviation or as a number (percentage)

Urban areas vs. rural areas, *p < 0.05, **p < 0.01, ***p < 0.001

BMI, body mass index; Alb, serum albumin level; LDL-C, low-density lipoprotein cholesterol level; CRE, Creatinine; eGFR, estimated glomerular filtration rate; SDSCA, the Summary of Diabetes Self-Care Activities Measure

The mean ages of the participants in urban and rural areas were 70.6 ± 5.5 and 69.0 ± 5.2 years, respectively. In addition, the proportion of women, mean body mass index, diabetes duration, HbA1c level, serum albumin level, and low-density lipoprotein cholesterol level in urban and rural areas were 48.0% and 43.2%, 24.1 ± 3.6 and 25.0 ± 4.0 kg/m^2^, 16.6 ± 10.9 and 12.0 ± 10.3 years, 7.3 ± 1.0% (57 ± 11 mmol/mol) and 7.0 ± 0.9% (53 ± 10 mmol/mol), 4.16 ± 0.31 and 4.30 ± 0.38 g/ml, and 103.7 ± 28.7 and 106.2 ± 26.6 mg/dl, respectively.

The proportion of insulin-treated older adults and the diet- and exercise-related SDSCA scores were higher in urban areas than in rural areas (*p* = 0.001, 0.003, and 0.036, respectively). The medication-related SDSCA score was significantly higher in rural areas than in urban areas (*p* = 0.001). Regarding diabetic complications, the prevalence of hypoglycemia, serious hypoglycemia, nephropathy, peripheral neuropathy, and peripheral artery disease was significantly higher in urban areas than in rural areas.

Among lifestyle-related factors, only the smoking habit rate was higher in rural areas than in urban areas (*p* = 0.005). Drinking habit rate, employment rate, education level, the proportion of people living alone, and irregular lifestyle were not significantly different between the regions.

### Prevalence and characteristics of frailty

Regional differences were observed in the prevalence of frailty, with robustness being particularly significantly lower in rural areas than in urban areas (29.7% vs. 43.9%, *p* = 0.018). The scores on the instrumental ADL (IADL) and social ADL (SADL) subscales of KCL were significantly higher in rural areas (*p* < 0.001, *p* = 0.029). Grip strength was also higher in rural areas (*p* = 0.001). No significant differences existed between the total and other subscale scores for KCL and body composition (Table 2).

**Table 2:** Body composition and frailty

Variables	Urban areas (*n* = 269)	Rural areas (*n* = 148)	*p*
Body fat mass (kg)	18.7 ± 5.2	18.9 ± 5.8	0.786
Skeletal muscle mass (kg)	16.2 ± 4.4	16.4 ± 3.9	0.598
Grip strength (kg)	26.8 ± 8.0	29.6 ± 8.2	0.001**
Frailty			0.018*
Frailty	50 (18.6)	34 (23.0)	
Prefrailty	101 (37.5)	70 (47.3)	
Robust	118 (43.9)^†^	44 (29.7)	
KCL score (frailty index)	4.9 ± 3.6	5.4 ± 3.2	0.160
KCL subscale score			
Instrumental ADL	0.3 ± 0.6	0.6 ± 0.9	< 0.001***
Social ADL	0.8 ± 0.9	1.0 ± 0.9	0.029*
Physical activities	1.3 ± 1.2	1.3 ± 1.2	0.818
Nutritional status	0.3 ± 0.5	0.3 ± 0.5	0.706
Oral function	0.9 ± 0.9	0.9 ± 0.9	0.605
Cognitive function	0.6 ± 0.8	0.5 ± 0.7	0.150
Depressive mood	0.7 ± 1.2	0.8 ± 1.2	0.545

Values in the table are presented as the mean ± standard deviation or as a number (percentage)

Urban areas vs. rural areas, *p < 0.05, **p < 0.01, ***p < 0.001. Residual analysis, ^†^*p* < 0.05

KCL, Kihon Checklist; ADL, Activities of Daily Living

### Relationship between frailty and region

The logistic regression analyses showed that frailty was significantly associated with the region (odds ratio [OR] = 2.55, 95% confidence interval [CI]: 1.38–4.71, *p* = 0.003) and HbA1c levels (OR = 1.45, 95% CI: 1.10–1.93, *p* = 0.010) after adjusting for age, gender, HbA1c levels, and diabetes duration. Pre-frailty was significantly associated with the region (OR = 2.10, 95% CI: 1.30–3.41, *p* = 0.003) (Table 3).

**Table 3:** Relationship between frailty and region

**< For frailty>**	**Model 1**			**Model 2**			**Model 3**	
	**OR (95% CI)**	***p***		**OR (95% CI)**	***p***		**OR (95% CI)**	***p***
Region (Rural areas)	2.035 (1.145–3.619)	0.015**		2.350 (1.294–4.267)	0.005**		2.554 (1.384–4.711)	0.003**
Age (years)	1.036 (0.984–1.091)	0.175		1.038 (0.985–1.094)	0.159		1.032 (0.978–1.089)	0.247
Gender (Women)	1.702 (0.988–2.931)	0.985		1.690 (0.970–2.944)	0.064		1.736 (0.991–3.036)	0.053
HbA1c (%)	-	-		1.487 (1.130–1.958)	0.005**		1.453 (1.095–1.926)	0.010*
Diabetes duration (years)	-	-		-	-		1.012 (0.984–1.041)	0.413
**< For prefrailty>**	**Model 1**			**Model 2**			**Model 3**	
	**OR (95% CI)**	***p***		**OR (95% CI)**	***p***		**OR (95% CI)**	***p***
Region (Rural areas)	1.834 (1.152–2.919)	0.011*		1.930 (1.204–3.096)	0.006**		2.102 (1.296–3.408)	0.003**
Age (years)	1.008 (0.968–1.050)	0.689		1.009 (0.969–1.051)	0.668		0.999 (0.957–1.042)	0.945
Gender (Women)	0.688 (0.443–1.068)	0.095		0.708 (0.455–1.101)	0.125		0.715 (0.457–1.118)	0.141
HbA1c (%)	-	-		1.140 (0.897–1.450)	0.284		1.096 (0.853–1.408)	0.473
Diabetes duration (years)	-	-		-	-		1.016 (0.994–1.038)	0.164

Values are presented as odds ratios (95% CI). *p < 0.05, **p < 0.01

Logistic regression analyses were performed for frailty and prefrailty after adjusting for age and gender (model 1); age, gender, and HbA1c (model 2); and age, gender, HbA1c, and diabetes duration (model 3)

OR, odds ratio; CI, confidence interval

### Relationship between subscale score of KCL and region

After adjusting for age, gender, HbA1c levels, and diabetes duration in the multiple linear regression analysis, regional differences were found in IADL and SADL but not in physical activities, nutritional status, oral function, cognitive function, and depressive mood (Table 4).

**Table 4:** Relationship between subscale scores of KCL and region

	**Instrumental ADL**			**Social ADL**			**Physical activities**			**Nutritional status**	
	**B (SE)**	***p***		**B (SE)**	***p***		**B (SE)**	***p***		**B (SE)**	***p***
Region (Rural areas)	0.279 (0.073)	< 0.001***		0.265 (0.097)	0.006**		0.146 (0.121)	0.227		0.005 (0.051)	0.919
Age (years)	0.012 (0.006)	0.067		0.006 (0.009)	0.453		0.024 (0.011)	0.025*		-0.015 (0.005)	< 0.001***
Gender (Women)	-0.437 (0.067)	< 0.001***		-0.211 (0.090)	0.020*		0.668 (0.112)	< 0.001***		-0.001 (0.048)	0.982
HbA1c (%)	0.019 (0.036)	0.599		0.091 (0.048)	0.058		0.172 (0.059)	0.004**		-0.020 (0.025)	0.439
Diabetes duration (years)	-0.002 (0.003)	0.514		0.005 (0.004)	0.271		0.009 (0.005)	0.099		0.001 (0.002)	0.631
	**Oral function**			**Cognitive function**			**Depressive mood**				
	**B (SE)**	***p***		**B (SE)**	***p***		**B (SE)**	***p***			
Region (Rural areas)	0.092 (0.094)	0.327		-0.078 (0.078)	0.317		0.162 (0.124)	0.194			
Age (years)	0.006 (0.008)	0.492		0.007 (0.007)	0.338		0.011 (0.011)	0.307			
Gender (Women)	0.308 (0.087)	< 0.001***		0.151 (0.073)	0.039*		0.371 (0.115)	0.001**			
HbA1c (%)	0.059 (0.046)	0.207		0.045 (0.039)	0.244		0.161 (0.061)	0.009**			
Diabetes duration (years)	0.000 (0.004)	0.938		-0.002 (0.004)	0.668		-0.001 (0.006)	0.885			

The values in the table are presented as partial regression coefficients (standard errors). *p < 0.05, **p < 0.01, ***p < 0.001

Multiple linear regression analyses were performed for the subscale scores of KCL adjusted for age, gender, HbA1c and diabetes duration

KCL, Kihon Checklist; ADL, activities of daily living; B, partial regression coefficient; SE, standard error

The IADL and SADL scores were significantly associated with region (rural areas) (B = 0.279, standard error [SE] = 0.073, *p* < 0.001; B = 0.265, SE = 0.097, *p* = 0.006) and gender (women) (B = − 0.437, SE = 0.067, *p* < 0.001; B = -0.211, SE = 0.090, *p* = 0.020), respectively.

Physical activity scores were significantly associated with age (B = 0.024, SE = 0.011, *p* = 0.025), gender (women) (B = 0.668, SE = 0.112, *p* < 0.001), and HbA1c levels (B = 0.172, SE = 0.059, *p* = 0.004). Nutritional status scores were significantly associated with age (B = -0.015, SE = 0.005, *p* < 0.001). Oral and cognitive function scores were significantly associated with gender (women) (B = 0.308, SE = 0.087, *p* < 0.001; B = 0.151, SE = 0.073, *p* = 0.039). Depressive mood was significantly associated with gender (women) (B = 0.371, SE = 0.115, *p* = 0.001) and HbA1c levels (B = 0.161, SE = 0.061, *p* = 0.009).

For reference, the responses to each question on the KCL were compared between the urban and rural areas (Table 5). Of 25 questions assessing frailty, implementation was significantly higher in urban areas than in rural areas for four items: “1. Do you go out by bus or train by yourself?” (90.7% vs. 67.6%, *p* < 0.001), “4. Do you sometimes visit your friends?” (68.4% vs. 57.4%, *p* = 0.025), “5. Do you turn to your family or friends for advice?” (84.0% vs. 75.7%, *p* = 0.038), and “8. Do you normally walk continuously for 15 minutes?” (90.7% vs. 82.4%, *p* = 0.014).

**Table 5:** Percentage of “yes” responses to each question on KCL

Questions	Urban areas (*n* = 269)	Rural areas (*n* = 148)	*p*
1. Do you go out by bus or train by yourself?	90.7	67.6	< 0.001***
2. Do you go shopping to buy daily necessities by yourself?	92.6	89.2	0.240
3. Do you manage your own deposits and savings at the bank?	84.8	82.4	0.536
4. Do you sometimes visit your friends?	68.4	57.4	0.025*
5. Do you turn to your family or friends for advice?	84.0	75.7	0.038*
6. Do you normally climb stairs without using handrail or wall for support?	63.2	62.8	0.942
7. Do you normally stand up from a chair without any aids?	82.9	81.8	0.769
8. Do you normally walk continuously for 15 min?	90.7	82.4	0.014*
9. Have you experienced a fall in the past year?	30.1	25.0	0.268
10. Do you have a fear of falling while walking?	37.9	30.6	0.136
11. Have you lost 2 kg or more in the past 6 months?	26.4	30.4	0.382
12. If BMI is less than 18.5, this item is scored (yes).	4.8	2.7	0.293
13. Do you have any difficulties eating tough foods compared to 6 months ago?	26.0	27.0	0.824
14. Have you chocked on your tea or soup recently?	27.1	34.5	0.118
15. Do you often experience having a dry mouth?	32.0	28.4	0.447
16. Do you go out at least once a week?	94.1	93.2	0.744
17. Do you go out less frequently compared to last year?	21.9	22.3	0.932
18. Do your family or your friends point out your memory loss? e.g., “You ask the same question over and over again.”	20.1	14.2	0.134
19. Do you make a call by looking up phone numbers?	86.2	87.2	0.793
20. Do you find yourself not knowing today’s date?	25.7	21.8	0.377
21. In the last 2 weeks have you felt a lack of fulfillment in your daily life?	14.1	18.2	0.267
22. In the last 2 weeks have you felt a lack of joy when doing the things, you used to enjoy?	8.9	8.8	0.962
23. In the last 2 weeks have you felt difficulty in doing what you could do easily before?	16.4	16.3	0.994
24. In the 2 weeks have you felt helpless?	13.0	11.5	0.652
25. In the 2 weeks have you felt tired without a reason?	19.3	24.3	0.232

Values in the table represent percentages

Urban vs. rural areas, *p < 0.05, ***p < 0.001

KCL, Kihon Checklist

## Discussion

The present study examined regional differences in frailty between urban and rural areas among older adults with diabetes who could independently perform basic ADLs. Results showed that living in rural areas was associated with frailty and pre-frailty. Frailty in rural areas was characterized by lower IADL and SADL scores. Lower IADL and SADL scores were also associated with gender (men) but not with age, diabetes duration, or HbA1c levels.

IADLs include activities necessary for social life, such as shopping, making phone calls, and using public transportation, and complex ADLs, such as washing clothes and cooking. SADLs include interactions with others and going out. The lower IADL and SADL scores in rural areas suggest a higher risk of social frailty in rural areas than in urban areas. The risk of mortality owing to social frailty was reported to be 2.69 times higher than that towing to physical frailty [29, 30]. Social frailty precedes and causes impaired cognitive, physical, and psychological functioning [31, 32]. Therefore, it is important to prevent social frailty in rural areas.

In contrast, age, diabetes duration, and HbA1c levels were not associated with a decline in IADLs and SADLs. HbA1c levels and diabetes duration have generally been associated with frailty in older adults with diabetes [19, 33, 34], although some studies have found no association [35]. Because a previous study showed a stronger association between HbA1c and prognosis in individuals with physical frailty [36], the association with social ADLs in individuals with independent ADLs, such as the participants in this study, was considered weak. Therefore, glycemic control alone may be insufficient to prevent frailty in rural areas. In addition, the participants in the present study regularly attended diabetes outpatient clinics. Therefore, healthcare professionals in rural areas need to know the risk of decreased social activity and assess and promote such activity, even among older adults with diabetes who independently perform ADLs and can maintain hospital visits.

Notably, many participants in rural areas did not use public transportation or walk for > 15 min. This indicates that cars are their primary means of transportation and that they have few opportunities to walk unless they are aware of them. Furthermore, many did not visit friends or advise family or friends. Therefore, they may have fewer opportunities to interact deeply with others and be more isolated.

The availability of facilities and services related to necessary daily living functions and social participation within walking and biking distances are considered related to the social activities of older adults [37, 38]. In rural areas, accessibility to train stations, stores, bank automated teller machines, community centers, parks, and other social amenities that facilitate outings (safe sidewalks, outdoor lights, restrooms, and benches) is likely lacking. Therefore, lifestyle interventions to overcome this environmental disadvantage should be included in diabetes self-management education programs.

Because hospital visits are an important opportunity to prevent frailty in older adults with diabetes, it is necessary to support them in safely increasing their activity by suggesting that they park in a remote parking lot to increase their walking distance, assess peripheral neuropathy as a risk factor for frailty, and suggest an appropriate exercise load based on an assessment of diabetic complications. Furthermore, peer support programs that involve extensive interaction with others and group sessions that allow people to experience exercise and diet therapy while making friends need to be actively incorporated into diabetes education programs for older adults.

In addition, one possible factor associated with the higher risk of frailty in rural areas may be differences in health literacy. Many reports associate health literacy with frailty, indicating that older adults who have difficulty acquiring information, making decisions, and taking action in various health-related daily life situations are more likely to become frail in the future [39–42]. Low health literacy is also associated with poor self-management ability [43], non-adherence to treatment [44], low use of preventive services such as cancer screening [45], higher hospital readmission rates [46], and an increased risk of overall mortality [47]. Participants in both areas were regularly examined by diabetologists and received support from certified diabetes educators (CDEs), certified nurse practitioners, and nutritionists, indicating no significant differences in diabetes care or patient education between the rural and urban areas. However, the rate of implementation of health behaviors, such as diet, exercise, and smoking cessation, was higher in urban areas than in rural areas. Since a systematic review comparing health literacy in urban and rural areas also reported higher health literacy in urban areas [48], it will be necessary to consider local health literacy levels in frailty prevention.

Given these regional differences, intervention programs that combine individualized risk-based frailty prevention, diabetes management, and online health communication strategies delivered across geographic and time constraints are necessary. Having a smartphone improves health literacy and social support in older adults, lowering the risk of frailty [49]. While aging is associated with lower health literacy [50], a large longitudinal cohort study in the United Kingdom reported that internet use and social engagement help maintain health literacy maintenance in older adults [51]. Therefore, improved health literacy and internet use are key elements in both diabetes self-care and frailty prevention in older adults with diabetes. There is currently little evidence of the effectiveness of information and communication technologies in preventing frailty in older adults; however, a previous report found that a web-delivered group exercise intervention improved physical function in older adults with type 2 diabetes [52]. Improvements in HbA1c levels, patient activation, and self-efficacy were reported in people with type 2 diabetes who participated in an online diabetes self-management program compared with a usual care control group [53]. Based on previously identified risk factors [19], establishing an online program that assesses the individual risk of frailty, suggests risk-based frailty prevention strategies, and provides support may be useful.

This study had some limitations. First, the self-reported survey items may have introduced some reporting bias. However, this effect was minimized because the assessment instruments were reliable and validated, and most items were administered by trained nurses responsible for collecting measurements and information from the medical records. Second, there is no precise information on whether participants have lived in each region their entire lives. However, most participants have attended the same hospital or clinic for several years to a decade or more. Therefore, it is likely that few have moved to widely different environments, such as rural and urban areas, at least during the older age period. Additionally, not all participants underwent routine evaluation for hypoglycemia, including blood glucose or continuous glucose monitoring, which may have led to overlooking asymptomatic hypoglycemia. Specifically, self-monitoring of blood glucose was mainly performed by patients treated with insulin or GLP-1 RA, as continuous glucose monitoring was uncommon. Consequently, hypoglycemia may have been overlooked in patients treated with oral therapy. However, less severe hypoglycemia can be confirmed by patient self-reporting [24, 54]. Therefore, in this study, all information related to hypoglycemia was obtained, and information bias was minimized by evaluating both blood glucose measurement and self-report findings. Finally, although this was a nationwide survey, not all areas were covered. Notably, areas without diabetologists or diabetes-specific medical staff were not surveyed. Further research covering areas with poor access to specialized medical services is required.

## Conclusions

The results of this study indicate that older adults with diabetes who can independently perform ADLs are at a higher risk of frailty in rural areas, as characterized by a decline in IADLs and SADLs. The social environment should be considered in risk assessments for frailty prevention. In addition to environmental adjustments based on individual risks, intervention programs should be designed to include communication strategies that enable care and social participation across different environments.

## Data Availability

The datasets used and/or analysed in this study are available from the corresponding authors upon reasonable request and with the approval of the Ethics Committee for the transfer of data.
